# Preparation of Extracellular Matrix Developed Using Porcine Articular Cartilage and *In Vitro* Feasibility Study of Porcine Articular Cartilage as an Anti-Adhesive Film

**DOI:** 10.3390/ma9010049

**Published:** 2016-01-14

**Authors:** Ji Hye Baek, Kyungsook Kim, Soon Sim Yang, Seung Hun Park, Bo Ram Song, Hee-Woong Yun, Sung In Jeong, Young Jick Kim, Byoung Hyun Min, Moon Suk Kim

**Affiliations:** 1 Department of Molecular Science and Technology, Ajou University, Suwon 443-749, Korea; bjh3636@ajou.ac.kr (J.H.B.); poeme85@naver.com (K.K.); gather486@hanmail.net (S.S.Y.); hpt88@ajou.ac.kr (S.H.P.); br8551@naver.com (B.R.S.); yuni_06@naver.com (H.-W.Y.); 2 Advanced Radiation Technology, Korea Atomic Energy Research Institute, Jeollabuk-do 580-185, Korea; sxj0620@gmail.com; 3 Cell Therapy Center, Ajou University Medical Center, Suwon 443-749, Korea; kyjkyj95@hanmail.net

**Keywords:** porcine articular cartilage, anti-adhesive film, cross-linking, thermal treatment, incubation

## Abstract

In this study, we examined whether porcine articular cartilage (PAC) is a suitable and effective anti-adhesive material. PAC, which contained no non-collagenous tissue components, was collected by mechanical manipulation and decellularization of porcine knee cartilage. The PAC film for use as an anti-adhesive barrier was easily shaped into various sizes using homemade silicone molds. The PAC film was cross-linked to study the usefulness of the anti-adhesive barrier shape. The cross-linked PAC (Cx-PAC) film showed more stable physical properties over extended periods compared to uncross-linked PAC (UnCx-PAC) film. To control the mechanical properties, C*x*-PAC film was thermally treated at 45 °C or 65 °C followed by incubation at room temperature. The Cx-PAC films exhibited varying enthalpies, ultimate tensile strength values, and contact angles before and after thermal treatment and after incubation at room temperature. Next, to examine the anti-adhesive properties, human umbilical vein endothelial cells (HUVECs) were cultured on Cx-PAC and thermal-treated Cx-PAC films. Scanning electron microscopy, fluorescence, and MTT assays showed that HUVECs were well adhered to the surface of the plate and proliferated, indicating no inhibition of the attachment and proliferation of HUVECs. In contrast, Cx-PAC and thermal-treated Cx-PAC exhibited little and/or no cell attachment and proliferation because of the inhibition effect on HUVECs. In conclusion, we successfully developed a Cx-PAC film with controllable mechanical properties that can be used as an anti-adhesive barrier.

## 1. Introduction

During and/or after surgery, intra-tissue adhesion can induce serious post-operative complications such as chronic pelvic pain, small-bowel obstruction and infarction. Thus, numerous anti-adhesive techniques have been developed to suppress intra-tissue adhesion and serious related complications [1].

Among anti-adhesive techniques, anti-adhesive barriers using several biomaterials physically prevent direct contact between injured- or surgical-tissue surfaces and surrounding normal tissues during the temporal or critical period [2]. Numerous biodegradable or non-degradable biomaterials have been developed as anti-adhesive materials, some of which have been extensively applied in human trials as commercial grades approved by the US Food and Drug Administration [3,4,5,6].

Currently, polysaccharide-based film or gel-type anti-adhesive barriers, such as sodium hyaluronate, gelatin, and carboxymethyl cellulose, have been extensively investigated and are commercially available for clinical utilization [3,4,5,6,7,8,9,10]. In addition, several extracellular matrices, such as collagen, have been investigated in preclinical animal studies as anti-adhesive barriers [11,12,13].

Additionally, recent research efforts have focused on the development of an alternative anti-adhesive barrier composed of effective anti-adhesive material [14,15]. Articular cartilage has emerged as a promising extracellular matrix candidate for various biomedical applications [16]. Recent studies have suggested that articular cartilage directly inhibited vessel formation in an animal model *in vivo* due to the anti-adhesive properties of the proteoglycan rich matrix [17,18,19,20,21]. These results appeared to be correlated with its ability to inhibit the adhesion and proliferation of endothelial cells. Thus, articular cartilage may have decreased the incidence or severity of adhesions post-operatively.

Nonetheless, articular cartilage has not been established as an alternative anti-adhesive barrier material for preventing intra-tissue adhesion. We hypothesized that porcine articular cartilage (PAC) is a suitable and effective anti-adhesive material. PAC powder was easily prepared by decellularization, digestion, and sterilization. Thus, as the first aim of this work, we focused on the preparation and feasibility of PAC film as an anti-adhesive barrier. The PAC film shape was easily prepared in various sizes by using a homemade silicone molder.

However, the prepared PAC film has several drawbacks, including its difficulty in handling because of its fragility as a film and easy solubilization in biological solution. Additionally, the anti-adhesive barrier must be maintained in its *in vivo* shape for a period. To overcome these limitations, we prepared a cross-linked PAC (Cx-PAC) film. Cx-PAC films have more stable physical properties compared to uncross-linked PAC (UnCx-PAC) film.

No previous studies have examined the use of Cx-PAC film as an anti-adhesive barrier. Thus, the second aim of this work was to determine whether Cx-PAC film can serve as an anti-adhesive barrier.

PAC is a complex collagen fiber-composite material. Generally, collagen fibrils can be denatured by heating, which changes the collagen fibril molecular conformation. These changes to the heated collagen fibrils may affect the mechanical properties. We predicted that thermal treatment of Cx-PAC film might be used to control the mechanical properties. Thus, the final aim of this work was to examine whether thermal-treated Cx-PAC film can be used as an anti-adhesive barrier with controllable mechanical properties. Answers to these aims will enable development of PAC biomaterials and Cx-PAC films with controllable mechanical properties for use as an anti-adhesive barrier.

## 2. Results and Discussion

### 2.1. Prepared Cx-PAC Film Retained Shape While UnCx-PAC Deformed

**Figure 1:**
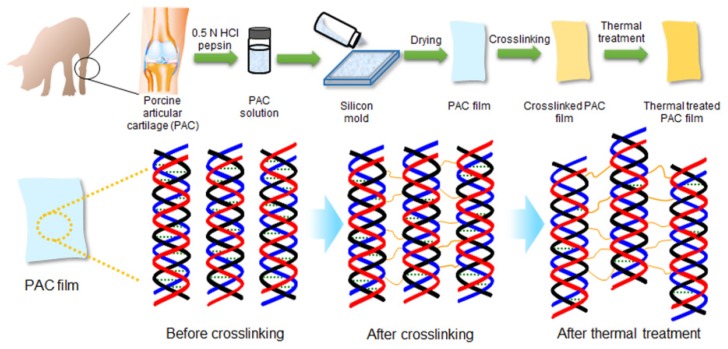
Schematic diagram for the preparation of UnCx-PAC films and thermal-treated Cx-PAC films.

A schematic diagram of PAC, UnCx-PAC film, and Cx-PAC film is shown in Figure 1. Degeneration of the structural properties of PAC must be avoided during the processing and manufacturing steps. First, PAC was collected from the porcine knee cartilage of the fore and hind legs. A fine powder of PAC was prepared after performing mechanical manipulation and decellularization of porcine knee cartilage. The PAC was macroscopically observed as a white color. The PAC showed no lacuna and cell nuclei after hematoxylin and eosin staining and no glycosaminoglycan and DNA contents, indicating the complete removal of all non-collagenous tissue components, blood, and cells (data not shown). Thus, the processing and manufacturing steps of porcine cartilage were applicable for the preparation of PAC.

However, the PAC swelled and was not soluble in biological solution. The water-insoluble PAC powder was solubilized by using an aqueous mixture solution of HCl and pepsin. The obtained PAC solution was added to the molders to form the PAC film after freeze-drying, but the PAC film did not maintain the film shape in water because it dissolved.

Therefore, to construct an UnCx-PAC film to a structure-remolded film shape, we chose glutaraldehyde to cross-link the PAC chains. Glutaraldehyde was reacted with the amine group on the intra- and inter-PAC chains. Unreacted glutaraldehyde was removed from the Cx-PAC film by washing with PBS. No glutaraldehyde was detected by high-performance liquid chromatography of Cx-PAC film (data not shown).

UnCx-PAC film did not maintain the film shape, while the prepared Cx-PAC film swelled and maintained the film shape, which was not soluble in biological solution. These results indicate that the manufactured Cx-PAC film can be used as an anti-adhesive barrier candidate.

### 2.2. Thermal Treatment Induced a Uniformed and Densely Organized Form of PAC Powder

**Figure 2:**
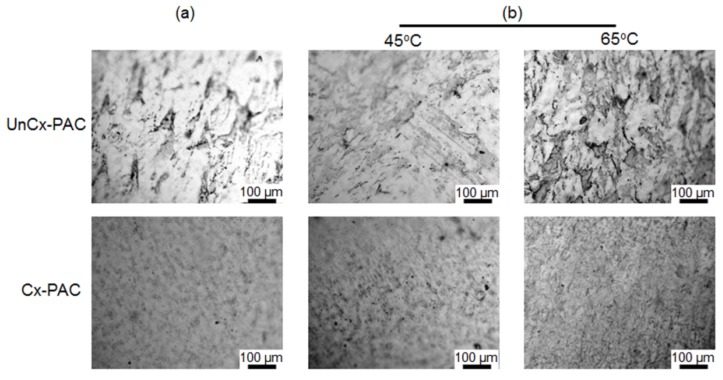
Optical images of UnCx-PAC and Cx-PAC film (**a**) before and (**b**) after thermal treatment at 45 °C and 65 °C.

To investigate the influence of heat on the PAC film by varying the heating temperatures and times, UnCx-PAC and Cx-PAC films were treated for 6 h, 12 h or 24 h at 45 °C or 65 °C. Figure 2 shows the optical images of UnCx-PAC and Cx-PAC films before and after thermal treatment. UnCx-PAC films exhibited agglomerated native PAC powder (black spots) regardless of the thermal treatment used. Cx-PAC films showed uniformed placement of PAC powder and densely organized placement images. These results indicate that the collagen in PAC was close in proximity because of the cross-linking between the glutaraldehyde and the amine groups within PAC, followed by partial integration between PAC chains.

### 2.3. Thermal Treatment Induced Change of Thermal, Mechanical and Surface Properties of Cx-PAC Films

**Figure 3:**
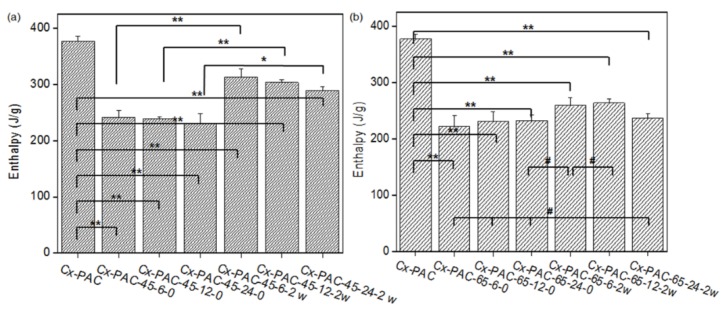
Enthalpy of Cx-PAC film aged zero and two weeks at room temperature for each Cx-PAC film before and after thermal treatment at (**a**) 45 °C and (**b**) 65 °C (* *p* < 0.05, ** *p* < 0.001, # *p* > 0.001).

Before and after the thermal treatments, Cx-PAC films were analyzed by differential scanning calorimetry (DSC). The Cx-PAC films exhibited broad and endothermic peaks indicating corresponding enthalpy changes (Figure 3). Cx-PAC showed a decreased enthalpy value (380 J/g) compared with UnCx-PAC (443 J/g).

The enthalpy of Cx-PAC films after thermal treatment at 45 °C or 65 °C decreased to 230–240 J/g. This indicated that the coiled collagen of Cx-PAC films underwent thermal denaturation [9]. Only a small enthalpy difference was observed between thermal treatments at 45 °C and 65 °C.

The thermal-treated Cx-PAC films were incubated at room temperature for two weeks. The enthalpy of Cx-PAC films treated at 45 °C increased to 290–310 J/g, while those treated at 65 °C slightly increased to 236–260 J/g. Specifically, Cx-PAC film treated at 65 °C showed a similar enthalpy value of 236 J/g compared with 232 J/g even before incubation at room temperature. The Cx-PAC films treated at 65 °C showed stronger denaturation of the coiled collagen of Cx-PAC films than the Cx-PAC films treated at 45 °C. These results indicate that the denaturation of collagen fibrils of Cx-PAC films require temperatures higher than 65 °C or a minimum heating time of 24 h. Thus, high heating temperatures and time are necessary to alter the collagen coil structure of Cx-PAC films.

**Figure 4:**
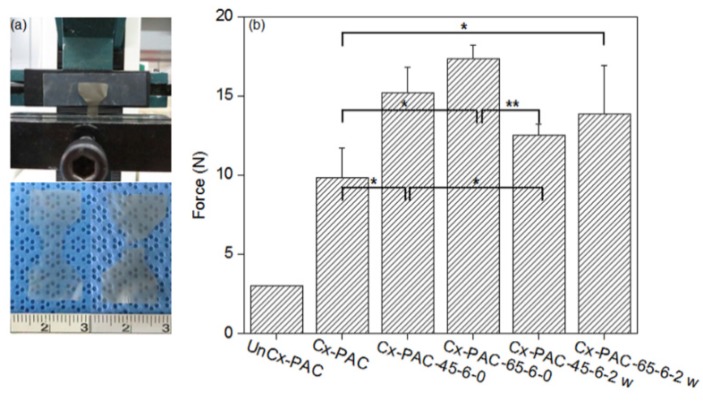
(**a**) Pictures of universal testing machine and film and (**b**) tensile strength of UnCx-PAC and Cx-PAC films aged for zero and two weeks at room temperature after thermal treatment at 45 °C and 65 °C (* *p* < 0.05, ** *p* < 0.001).

Figure 4a shows ultimate tensile strength measured using a universal testing machine and images of the dumbbell shape of Cx-PAC films. As shown in Figure 4b, the tensile strength of UnCx-PAC was 3 N. The ultimate tensile strength of Cx-PAC was increased significantly to 10 N. This result demonstrated that PAC chains were cross-linked by glutaraldehyde with the amine group on the intra- and inter-PAC chains.

Furthermore, thermal-treated Cx-PAC showed a significant increase to 15–17 N of the ultimate tensile strengths as compared with UnCx-PAC and Cx-PAC. In addition, the Cx-PAC treated at 65 °C resulted in a greater increase in ultimate tensile strength compared to that of Cx-PAC treated at 45 °C. The ultimate tensile strength value of thermal-treated Cx-PAC films was clearly increased by the thermal denaturation of the coiled collagen of Cx-PAC films: higher temperature treatment resulted in a higher increase in the ultimate tensile strength value.

When thermal-treated Cx-PAC films were incubated at room temperature for two weeks, the ultimate tensile strength significantly decreased to 12–13 N for Cx-PAC treated at 45 °C and 65 °C. However, the ultimate tensile strength values of Cx-PAC treated at 45 °C and 65 °C were higher than that of Cx-PAC before thermal treatment.

**Figure 5:**
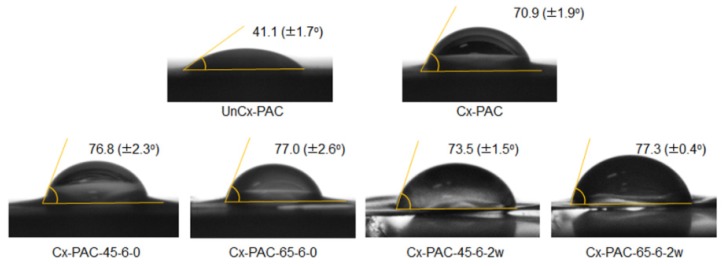
Contact angles of UnCx-PAC and Cx-PAC films aged for zero and two weeks at room temperature after thermal treatment at 45 °C and 65 °C.

The contact angles of UnCx-PAC and Cx-PAC were measured to examine film surface properties (Figure 5). UnCx-PAC showed contact angles of 41°. The contact angles of Cx-PAC increased to 71°. Thermal treatment resulted in an increase in contact angles to 77°–78°. The contact angles of Cx-PAC treated at 65 °C were maintained at similar values to before and after thermal treatment. Additionally, there was a slight decrease from 77° to 74° in the contact angles of Cx-PAC treated at 45 °C. This result indicated that thermal treatment of Cx-PAC could control the film surface properties.

### 2.4. Cx-PAC Films Significantly Inhibits the Attachment and Proliferation of HUVECs

Human umbilical vein endothelial cells (HUVECs) are widely used as an experimental model of anti-adhesive study. Thus, we evaluated the attachment and proliferation of HUVECs on Cx-PAC and thermal-treated Cx-PAC films compared with a plate as a control. The HUVECs cultured on Cx-PAC and thermal-treated Cx-PAC films were observed by SEM and fluorescence microscopy.

**Figure 6:**
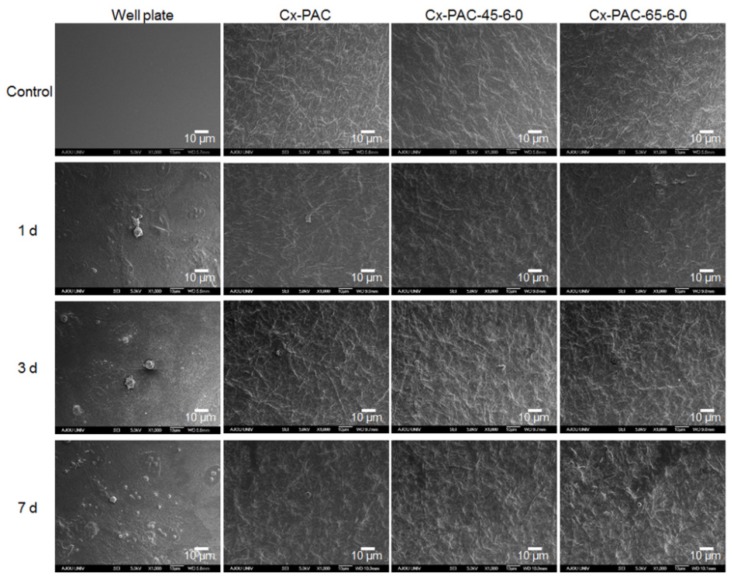
SEM micrographs showing no cell (control) and HUVECs cultured for one to seven days on plate and Cx-PAC film after thermal treatment at 45 °C and 65 °C. Magnification: 1000× and scale bar represents 10 μm.

As shown in Figure 6, in the SEM image, the plate showed smooth surface morphology, while the Cx-PAC and thermal-treated Cx-PAC films showed rough morphology, providing a suitable environment for the attachment and growth of the cells on films.

At one, three and seven days after incubation, HUVEC seeding studies showed that HUVECs adhered to the surface of the plate and proliferated effectively, indicating no inhibition of the attachment and proliferation of HUVECs. In contrast, Cx-PAC and thermal-treated Cx-PAC exhibited little and/or no cell attachment and proliferation by an obvious inhibitory effect of Cx-PAC films.

**Figure 7:**
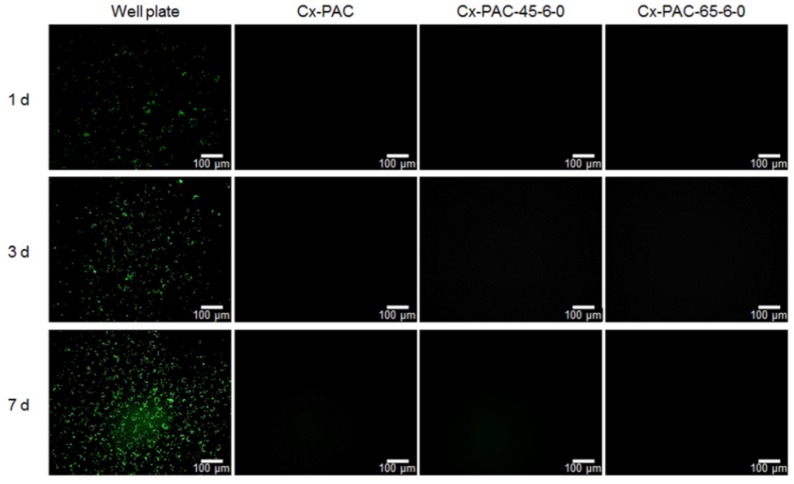
Fluorescent image showing PKH67-labeled HUVECs cultured for one to seven days on well plate and Cx-PAC film after thermal treatment at 45 °C and 65 °C. Magnification: 200× and scale bar represents 100 μm.

As shown in fluorescence images of PKH67-labeled HUVECs in Figure 7, the number of green dots, assignable to PKH67-labeled HUVECs, increased with increasing incubation time. However, there was little and/or no observation on Cx-PAC and thermal-treated Cx-PAC because of the inhibition effect.

**Figure 8:**
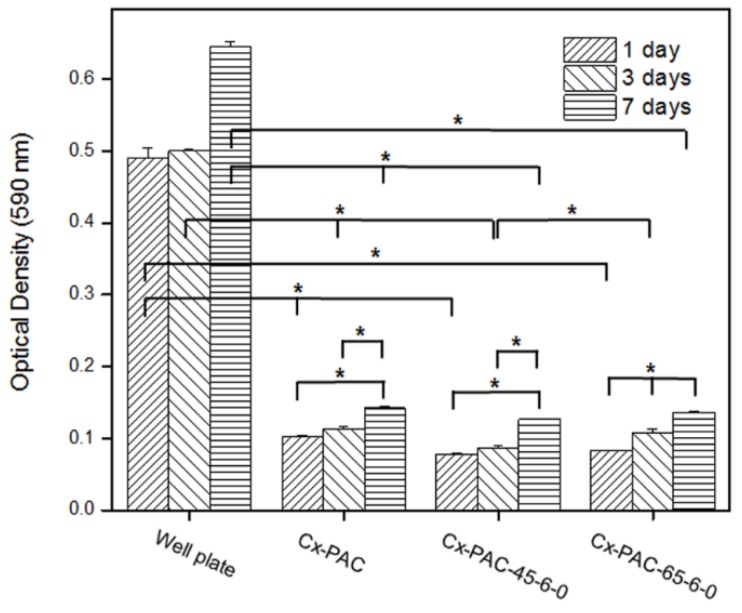
MTT assay of HUVECs cultured for one to seven days on well plates and Cx-PAC films after thermal treatment at 4 °C and 65 °C (* *p* < 0.001).

As shown in Figure 8, HUVEC seeding studies were evaluated using the MTT assay. Quantitative MTT analysis for Cx-PAC and thermal-treated Cx-PAC revealed a significant (*p* < 0.001) decrease in the optical density level compared with the plate after one, three and seven days of incubation. This indicated that Cx-PAC had approximately five- to seven-fold higher inhibition compared to the plate. Quantitative MTT analysis of the different Cx-PAC and thermal-treated Cx-PAC did not vary significantly. This result indicates that Cx-PAC significantly inhibits the attachment and proliferation of HUVECs.

## 3. Materials and Methods

### 3.1. Preparation of UnCx-PAC and Cx-PAC Film

Sections of PAC were harvested from market pigs (Finish pig, F1; Landrace + Yorkshire, around 100 kg at six months of age). Briefly, the fore and hind legs were used to prepare the PAC under sterile conditions. Non-cartilaginous tissues adhered on the fore and hind legs were dissected and washed with phosphate-buffered saline (PBS). The primitive PAC tissues were trimmed using a blade and washed several times with PBS. For decellularization, the resultant PAC tissues were treated with hypotonic buffer (10 mM Tris-HCl, pH 8.0) for 4 h at 4 °C and centrifuged for 10 min at 3300 rcf. Next, PAC tissues were stirred for 2 h at 4 °C in 1% sodium dodecyl sulfate in Tris-buffered saline (10 mM NaCl, pH 7.6) and washed five times with deionized water (DW) for 10 min at 3300 rcf. After washing, the resultant tissues were incubated in the presence of DNase (100 U/μm, Elpis Biotech, Daejeon, Korea) for 12 h at 37 °C. After an additional centrifugation step, the resulting tissues were washed in deionized water and then freeze-dried at −50 °C for at least four days to obtain water-insoluble PAC powder.

The prepared PAC powder was added to 10 mL vials containing an aqueous solution consisting of 0.5 M pepsin and then stirred for 24 h, followed by neutralization (pH 7.4) with 4 N NaOH solution. The PAC solution was dialyzed for 24 h with DW, which was replaced at 4 h intervals and freeze-dried at −50 °C for at least four days to yield the final water-soluble PAC powder. The PAC solution was prepared at 1.3 wt % concentration in DW. The PAC solution was carefully poured into a homemade silicone molder (30 × 30 mm^2^) to form the PAC film shape, followed by freeze-drying at −50 °C for at least four days. The PAC film was cross-linked with 0.1% glutaraldehyde concentrations for 1 h. The cross-linked PAC film was washed several times with DW to remove the unreacted glutaraldehyde, followed by freeze-drying at −50 °C for at least four days to give finally cross-linked PAC film.

### 3.2. Thermal Treatment of UnCx-PAC and Cx-PAC Films

UnCx-PAC and Cx-PAC films were thermally treated for 6 h, 12 h or 24 h at 45 °C or 65 °C in a vacuum oven (VO-27, Han Yang Scientific Equipment, Seoul, Korea). After the thermal treatments, the samples were allowed to cool to room temperature. The thermal-treated UnCx-PAC and Cx-PAC films were characterized within 3 h or after two-week incubation at room temperature after thermal treatment for 6 h, 12 h or 24 h at 45 °C or 65 °C. Each thermal-treated Cx-PAC film was assigned to Cx-PAC-(thermal treatment temperature)-(thermal treatment time)-(incubation time at room temperature after thermal treatment). All experiments were performed at least three times and the results were presented with mean and standard deviation (SD).

### 3.3. Characterization of Thermal-Treated UnCx-PAC and Cx-PAC Films

The heat of fusion (*ΔH*_m_) of Cx-PAC films without and with thermal treatment (*N* = 3) for 6 h, 12 h or 24 h at 45 °C or 65 °C was determined by differential scanning calorimetry (DSC; Q 10, TA Instruments, Eschborn, Germany) performed from −50 °C to 200 °C at a heating rate of 10 °C/min under a nitrogen atmosphere.

The water contact angle of Cx-PAC films was measured using the sessile drop method at room temperature with an optical bench-type contact angle goniometer (Phoenix 150, Suwon, Korea). One drop of purified water (10 μL) was deposited onto the UnCx-PAC, Cx-PAC and thermal-treated Cx-PAC films surface using a microsyringe attached to the goniometer. The water contact angle was measured within 5 s. The contact angle images were visualized under a CCTV camera (XC-75, SONY, Tokyo, Japan) equipped with Image J software (National Institutes of Health, Bethesda, MD, USA). The contact angles of three specimens were individually measured for each specimen and then pooled to obtain an average value.

The tensile strength of UnCx-PAC, Cx-PAC and thermal-treated Cx-PAC films were measured using a Universal Testing Machine (H5KT, Tinius-Olsen, Horsham, PA, USA). UnCx-PAC and Cx-PAC films were prepared in a dumbbell shape with sizes of 15 × 30 mm^2^ overall and 10 × 10 mm^2^ in the gauge area. The UnCx-PAC and Cx-PAC films were gripped at each end on the gauge area and pulled vertically at a rate of 0.5 mm/min using a 50 N load cell until the specimen was broken. All experiments were performed at least three times and the results were presented with mean and standard deviation (SD).

### 3.4. HUVECs Culture on Cx-PAC and Thermal-Treated Cx-PAC Films

First, human umbilical vein endothelial cells (HUVECs, InnoPharmaScreen, Asan, Korea) were labeled using the PKH67 Fluorescent Cell Linker Kit (Sigma, St. Louis, MO, USA) according to the manufacturer’s instructions. Briefly, the cultured HUVECs were washed with serum-free media and centrifuged for 5 min at 400× *g*. The provided diluent C (500 μL) was added to 1.5 × 10^5^ HUVECs and immediately mixed with 500 μL of PKH67 stock solution (4 × 10^−6^ M) in diluent C. After incubation for 5 min at room temperature, 1 mL of FBS was added and then the samples were incubated for 1 min to stop the labeling reaction. Finally, the HUVECs were pelleted for 5 min at 400× *g*, transferred to a fresh tube, and washed three times with HUVEC growth medium supplemented with 10 mL FBS, 0.2 mL hydrocortisone, 2 mL hFGF-B, 0.5 mL VEGF, 0.5 mL R3-IGF-1, 0.5 mL ascorbic acid, 0.5 mL hEGF, 0.5 mL FA-1000, and 0.5 mL heparin (EGM-2 SingleQuot Kit, Lonza, Basel, Switzerland).

Cx-PAC and thermal-treated Cx-PAC films were sterilized using ethylene oxide gas. For HUVEC culture experiments, Cx-PAC and thermal-treated Cx-PAC films with 12 mm diameters were prepared and placed individually into the wells of a 24-well tissue culture plate (Falcon, Pittsburgh, PA, USA) and then incubated for 4 h in culture media. After removing the media by suction, HUVECs (1 × 10^5^ cells/well) were transferred to each well and incubated for one, three and seven days. The culture media was changed every day throughout the studies. All experiments were performed at least three times and the results were presented with mean and standard deviation (SD).

### 3.5. Anti-Adhesive Properties of Cx-PAC Films

The morphology of HUVECs in the well plate, Cx-PAC and thermal-treated Cx-PAC films were examined using scanning electron microscopy (SEM) with a JSM-6380 SEM (JEOL, Tokyo, Japan). The HUVECs (1 × 10^5^ cells/well) on the well plate, Cx-PAC and thermal-treated Cx-PAC films were removed after one, three and seven days and fixed with 10% formaldehyde (Sigma) for 1 h, followed by dehydration using a series of 60, 70, 80, 90, and 100 of ethyl alcohol. The fixed HUVECs on the well plate, Cx-PAC and thermal-treated Cx-PAC films were coated with a conductive layer of gold using a plasma-sputtering apparatus (PS-1200; Para One, Seoul, Korea) and SEM (JSM-6700F, JEOL, Peabody, MA, USA) images were obtained.

For fluorescence imaging of HUVECs on the well plate, Cx-PAC and thermal-treated Cx-PAC films’ fluorescence were visualized under an inverted phase-contrast microscope (Eclipse TS100, Nikon, Tokyo, Japan). For the MTT assay, HUVEC viability in three well plates, Cx-PAC and thermal-treated Cx-PAC films evaluated individually and then the average value was calculated. Briefly, 100 mL of PBS solution of the MTT tetrazolium substrate (100 μL, Roche, Basel, Switzerland) was added after one, three and seven days. After incubation for 30 min at 37 °C, the resulting violet formazan precipitate was solubilized by the addition of 500 μL of DMSO and shaking for 30 min. An aliquot from each well (100 μL) was transferred to a 96-well plate. The solutions were then read using a ELISA plate reader (EL808 ultra microplate reader; Bio-Tek, Winooski, VT, USA). The optical density of each well was determined at 590 nm.

### 3.6. Statistical Analysis

DSC data and tensile strength results for the UnCx-PAC, Cx-PAC and thermal-treated Cx-PAC films were evaluated using independent experiments with *n* = 3 for each data point. The MTT assay of the PAC films was performed using independent experiments with *n* = 9 for each data point. All data were given as the mean and standard deviation (SD) values. The results were analyzed using one-way ANOVA with Bonferroni’s *post-hoc* tests. All statistical analysis was carried out using SPSS version 12.0 statistical software (SPSS, Inc., Chicago, IL, USA).

## 4. Conclusions

Here, we explored the potential utility of the PAC biomaterial as an anti-adhesive barrier. A Cx-PAC film was easily prepared from PAC biomaterial. We successfully developed a feasible Cx-PAC film with controllable properties as a useful experimental biomaterial platform for anti-adhesive barrier applications. Further experiments are necessary to investigate the *in vivo* feasibility utility in animal models.

## References

[1] Robb W.B., Mariette C. (2014). Strategies in the prevention of the formation of postoperative adhesions in digestive surgery: A systematic review of the literature. Dis. Colon Rectum.

[2] Qiu Y., Zhang N., An Y.H., Wen X. (2007). Biomaterial strategies to reduce implant-associated infections. Int. J. Artif. Organs.

[3] Matsuda S., Se N., Iwata H., Ikada Y. (2002). Evaluation of the antiadhesion potential of UV cross-linked gelatin films in a rat abdominal model. Biomaterials.

[4] Sakuma K., Iguchi A., Ikada Y., Tabayashi K. (2005). Closure of the pericardium using synthetic bioabsorbable polymers. Ann. Thorac. Surg..

[5] Burns J.W., Colt M.J., Burgees L.S., Skinner K.C. (1997). Preclinical evaluation of seprafilm bioresorbable membrane. Eur. J. Surg. Suppl..

[6] Karygianni L., Jähnig A., Schienle S., Bernsmann F., Adolfsson E., Kohal R.J., Chevalier J., Hellwig E., Al-Ahmad A. (2013). Initial bacterial adhesion on different yttria-stabilized tetragonal zirconia implant surfaces *in vitro*. Materials.

[7] Becker J.M., Dayton M.T., Fazio V.W., Beck D.E., Stryker S.J., Wexner S.D., Wolff B.G., Roberts P.L., Smith L.E., Sweeney S.A. (1996). Prevention of postoperative abdominal adhesions by a sodium hyaluronate-based bioresorbable membrane: A prospective randomized double-blind multicenter study. J. Am. Coll. Surg..

[8] Choi K.-H., Song B.R., Choi B.H., Lee M., Park S.R., Min B.-H. (2012). Cartilage tissue engineering using chondrocyte-derived extracellular matrix scaffold suppressed vessel invasion during chondrogenesis of mesenchymal stem cells *in vivo*. Tissue Eng. Regen. Med..

[9] Tsujimoto H., Tanzawa A., Matoba M., Hashimoto A., Suzuki S., Morita S., Ikada Y., Hagiwara A. (2013). The antiadhesive effect of thermally cross-linked gelatin film and its influence on the intestinal anastomosis in canine models. J. Biomed. Mater. Res. B Appl. Biomater..

[10] Lee S.Y., Bang S., Kim S., Jo S.Y., Kim B., Hwang Y., Noh I. (2015). Synthesis and *in vitro* characterizations of porous carboxymethyl cellulose-poly(ethylene oxide) hydrogel film. Biomater. Res..

[11] Tang S., Yang W., Mao X. (2007). Agarose/collagen composite scaffold as an anti-adhesive sheet. Biomed. Mater..

[12] Suwa Y., Nam K., Ozeki K., Kimura T., Kishida A., Masuzawa T. (2015). Thermal denaturation behavior of collagen fibrils in wet and dry environment. J. Biomed. Mater. Res. B Appl. Biomater..

[13] Yang J.W., Heo M.S., Lee C.H., Moon S.W., Min B.H., Choi B.H., Kang M.S., Moon S.H. (2015). The effect of the cell-derived extracellular matrix membrane on wound adhesions in rabbit strabismus. Tissue Eng. Regen. Med..

[14] Tsujimoto H., Tanzawa A., Miyamoto H., Horii T., Tsuji M., Kawasumi A., Tamura A., Wang Z., Abe R., Tanaka S. (2015). Biological properties of a thermally crosslinked gelatin film as a novel anti-adhesive material: Relationship between the biological properties and the extent of thermal crosslinking. J. Biomed. Mater. Res. B Appl. Biomater..

[15] Umeki S., Suzuki R., Ema Y., Shimojima M., Nishimura Y., Okuda M., Mizuno T. (2013). Anti-adhesive property of P-selectin glycoprotein ligand-1 (PSGL-1) due to steric hindrance effect. J. Cell Biochem..

[16] Doulabi A.H., Mequanint K., Mohammadi H. (2014). Blends and nanocomposite biomaterials for articular cartilage tissue engineering. Materials.

[17] Li T.Z., Jin C.Z., Choi B.H., Kim M.S., Kim Y.J., Park S.R., Yoon J.H., Min B.-H. (2012). Using cartilage extracellular matrix (CECM) membrane to enhance the reparability of the bone marrow stimulation technique for articular cartilage defect in canine model. Adv. Funct. Mater..

[18] Huey D.J., Hu J.C., Athanasiou K.A. (2012). Unlike bone, cartilage regeneration remains elusive. Science.

[19] Lee J.C., Min H.J., Lee S., Seong S.C., Lee M.C. (2013). Effect of chondroitinase ABC on adhesion and behavior of synovial membrane-derived mesenchymal stem cells in rabbit partial-thickness chondral defects. J. Orthop. Res..

[20] Chen C., Liu J.M., Chua C., Chou S., Shyu V.B., Chen J. (2014). Cartilage tissue engineering with silk fibroin scaffolds fabricated by indirect additive manufacturing technology. Materials.

[21] Bara J.J., Johnson W.E., Caterson B., Roberts S. (2012). Articular cartilage glycosaminoglycans inhibit the adhesion of endothelial cells. Connect. Tissue Res..

